# The leptin fragment Lep116-130 attenuates hedonic consumption and sucrose-seeking in mice

**DOI:** 10.3389/fphar.2026.1748508

**Published:** 2026-03-24

**Authors:** R. Ramírez-Romero, Y. Jiménez, B. Kerr, L. Dinamarca-Villarroel, A. Robles, P. Llanos, P. Montaña, V. Cortés, C. Perez-Leighton, R. Baudrand

**Affiliations:** 1 Facultad de Ciencias Biológicas, Pontificia Universidad Católica de Chile, Santiago, Chile; 2 Centro de Biología Celular y Biomedicina-CEBICEM, Facultad de Ciencias, Universidad San Sebastián, Santiago, Chile; 3 Facultad de Química y Farmacias, Pontificia Universidad Católica de Chile, Santiago, Chile; 4 Centro de Estudios en Ejercicio, Metabolismo y Cáncer, Facultad de Medicina, Universidad de Chile, Santiago, Chile; 5 Facultad de Odontología, Instituto de Investigación en Ciencias Odontológicas, Universidad de Chile, Santiago, Chile; 6 Departamento de Nutrición, Diabetes y Metabolismo, Facultad de Medicina, Pontificia Universidad Católica de Chile, Santiago, Chile; 7 Departamento de Endocrinología, Facultad de Medicina, Pontificia Universidad Católica de Chile, Santiago, Chile; 8 Centro Translacional de Endocrinología (CETREN), Facultad de Medicina, Pontificia Universidad Católica de Chile, Santiago, Chile

**Keywords:** food intake, hedonic intake, LepRb, leptin, Leptin116-130, sucrose motivation

## Abstract

**Background:**

Increased hedonic intake of palatable and high-calorie foods is one of the leading causes of obesity and is present in eating disorders. Thus, identifying new tools to manipulate hedonic intake could open new treatments for disorders of hunger and energy balance. Leptin 116–130 (Lep_116-130_) is a biologically active fragment of leptin, an anorectic hormone essential for controlling energy balance. Lep_116-130_ can reduce the intake of standard food in rodents, but its mechanism of action remains poorly understood, and whether it reduces hedonic intake has not been tested.

**Methods:**

*In silico* molecular docking was performed to determine the binding of Lep_116-130_ to the long isoform of leptin receptor (LepRb). *In vitro* cellular assays were performed to determine whether Lep_116-130_ increased intracellular calcium levels. Behavioral feeding tests were used to characterize the intake of standard and palatable, high-calorie food under acute and chronic administration of Lep_116-130_ to leptin-deficient (ob/ob) and wild-type mice.

**Results:**

*In silico* assays suggest Lep_116-130_ was less likely to bind to LepRb than leptin, and *in vitro* assays showed that Lep_116-130_ does not increase intracellular calcium in a hypothalamic cell line that expresses LepRb. The *in vivo* acute administration of Lep_116-130_ did not reduce standard food intake in wild-type or *ob*/*ob* mice, but it decreased intake of high-sucrose chocolate-flavored pellets in *ob*/*ob* mice. Chronic administration of Lep_116-130_ did not reduce body weight, whereas it prevented increased sucrose consumption after repeated sucrose demand tests exclusively in *ob/ob* mice.

**Conclusion:**

Lep_116-130_ can regulate hedonic food consumption in obese mice, likely without a major contribution of LepRb binding, providing a proof-of-concept of Lep^116-130^ as a modulator of reward-driven intake.

## 1 Introduction

Food intake is a motivated behavior that is essential for survival. The neuroendocrine control of food intake involves sensing the severity of fasting to increase motivation to seek and consume food, and this drive is reduced in the postprandial state (Stuber and Wise, 2016; Rossi and Stuber, 2018; Morton et al., 2014). This system is proposed to have evolved in an environment of food scarcity (Ulijaszek, 2002; James et al., 2019), which explains why humans and other animals exhibit a higher motivation to seek and experience greater pleasure when consuming energy-dense foods (Berthoud et al., 2017). While this innate trait may increase the chances of survival in an environment with limited availability of high-calorie foods (Krebs, 2009), the excessive availability of these foods in most modern societies drives their intake, leading to pleasure-seeking behavior regardless of fasting or satiety (Krebs, 2009; Drewnowski et al., 2020; Collaborators, 2019). This behavior, known as hedonic intake (Stuber and Wise, 2016; Rossi and Stuber, 2018; Morton et al., 2014) is proposed to be a major driver of obesity (Krebs, 2009; Drewnowski et al., 2020), a disease with 16% worldwide prevalence (Collaboration, 2024), and is also present in eating disorders such as binge-eating disorder (Witt et al., 2014). In recent years there has been a surge in new pharmacological therapies to treat obesity by reducing hedonic intake (McGowan et al., 2025; Shi et al., 2024). However, the limitations and side effects of these drugs (mainly gastrointestinal) (Liu et al., 2025) and the limited number of drugs to treat hedonic intake in eating disorders (Davis and Attia, 2017) indicate there is still a need for new pharmacological tools to treat disorders associated with a dysregulated control of hedonic intake.

Leptin 116–130 (Lep_116-130_) is a 15 aa synthetic peptide generated by the enzymatic cleavage of the peptide hormone leptin (Grasso et al., 1997). Leptin is released by adipocytes and, upon reaching the brain, alters neuronal activity in the hypothalamus (and other brain regions) thereby reducing food-seeking behavior and hunger, ultimately leading to decreased food intake (Kwon et al., 2016; Lee et al., 2023; Skoracka et al., 2025). Consequently, leptin-deficient mice (*ob*/*ob*) or humans with leptin deficiency exhibit hyperphagia and accumulate excessive white adipose tissue, alongside reduced glucose tolerance and insulin sensitivity (Park and Ahima, 2015; Koch et al., 2010; Ahima et al., 1996), dysfunctions rescued by leptin supplementation (Halaas et al., 1995; Wong et al., 2016). Originally, Lep_116-130_ was synthesized in the pursuit of finding leptin fragments that mimic leptin’s biological effects (Grasso et al., 1997). Lep_116-130_ can reduce food intake and body weight, as repeated intraperitoneal (IP) administration of Lep_116-130_ to female *ob*/*ob* mice (1 mg/day, once daily) reduced food intake and body weight starting after 7 days of treatment (Grasso et al., 1997; Grasso et al., 1999a). Lep_116-130_ also mimics other effects of leptin, such as increasing episodic-like memory performance in wild-type mice, reducing plasma adrenocorticotrophin hormone and corticosterone levels, and increasing the secretion of luteinizing hormone and prolactin in rats (Malekizadeh et al., 2017; Malendowicz et al., 2000; Markowska et al., 2004; Gonzalez et al., 1999). Nevertheless, Lep_116-130_ fails to mimic other reported biological effects of leptin, such as reducing plasma aldosterone concentration (Malendowicz et al., 2000; Grasso et al., 1999b). Overall, these studies suggest that Lep_116-130_ mimics some, but not all, of the biological effects described for leptin. Further, whether Lep_116-130_ can reduce hedonic intake or alter motivational aspects of eating behavior has not been tested.

The mechanisms underlying the action of Lep_116-130_ are poorly understood. In cells transfected with the long isoform of leptin receptor (LepRb), Lep_116-130_ was unable to trigger the canonical phosphorylation cascade leading to activation of the transcription factor STAT3 or compete for the binding of labeled leptin to LepRb (Grasso et al., 1999b). Also, the IP administration of Lep_116-130_ to female leptin receptor-deficient (*db*/*db)* mice (1 mg/day, once daily for 7 days) reduced body weight without changes in food intake (Grasso et al., 1999b), suggesting that Lep_116-130_ can activate non-LepRb-dependent signaling pathways. However, Lep_116-130_ can activate STAT3 in a neuroblastoma cell line known to express LepRb (Malekizadeh et al., 2017; Benomar et al., 2005; Russo et al., 2004), and, in hippocampal brain slices (a brain region known to express functional LepRb) (Hamilton and Harvey, 2021), Lep_116-130_ has similar effects on synaptic function to those of leptin (Malekizadeh et al., 2017), suggesting that, under certain conditions, Lep_116-130_ might bind and activate LepRb. Thus, whether the biological effects of Lep_116-130_ are mediated by LepRb remains unclear.

Here, we aimed to better understand the cellular mechanisms of action of Lep_116-113_ and determine whether this peptide could regulate hedonic intake. To gain insights into its mechanism of action, we used molecular docking to examine whether Lep_116-130_ binds to LepRb, cellular assays to examine whether Lep_116-130_ alters intracellular calcium signaling in hypothalamic cells, and tested whether Lep_116-130_ can reduce intake of standard rodent food and hedonic intake under conditions of free and operant access to energy-dense foods.

## 2 Methods

### 2.1 Animals

Male and female leptin-deficient mice (*ob*/*ob)* and their wild-type littermates were generated by breeding heterozygous (*ob*/+) parents. Original breeders were purchased from The Jackson Laboratory (Bar Harbor, ME, United States, B6.Cg-Lepob/J, #000632). A total of 21 ob/ob mice (body weight: 53.07 ± 1.51 g) and 18 wild-type mice (body weight: 26.59 ± 0.92 g) were used in this study. Mice were single-housed for 1 week before the start of the experiments (8–12 weeks old, 21 °C–23 °C, with a 12:12-h light–dark cycle) in clear, bottom cages with corncob bedding and environmental enrichment materials with *ad libitum* access to food (ProLab RMH-3000, Lab Diets, United States; 25.96% kcal from protein, 14.93% from fat, and 59.11% from carbohydrate) and water unless otherwise noted. All experimental procedures were approved by the Institutional Animal Care and Use Committee of the Pontificia Universidad Católica de Chile.

### 2.2 Pharmacological agents

For IP injections, Lep_116-130_ (Mimotopes, #55148-002) or Exendin-4 (E4, Tocris, #1933) were prepared in saline (NaCl 0.9%), aliquoted for single use, and stored at −20 °C. For intracellular calcium imaging, Lep_116-130_ and mouse recombinant leptin (#L3772-1 MG, Sigma-Aldrich) were prepared in imaging medium (Ozcan et al., 2015), aliquoted, and stored at −80 °C before use. For mini-osmotic pump delivery, Lep_116-130_ was prepared in sterile PBS (NaCl 136 mM; KCl 2.6 mM; Na_2_HPO_4_ 10.2 mM; KH_2_PO_4_ 1.8 mM) and used immediately.

### 2.3 Molecular docking of Lep_116-130_ and LepRb

The structure of mouse LepRb was extracted from the Protein Data Bank (PDB 8DH8). The protein has no mutations and was resolved with leptin bound in its central site (Saxton et al., 2023). As a control, we included mouse leptin, which was modeled using human leptin (PDB 1AX8) because the protein structures for mouse leptin (GenBank ADM72802.1) did not include all residues. Using Modeler 10.2 (Webb and Sali, 2016) we generated 100 models to construct the side chains of the LepRb and leptin and relax their protein structures. For LepRb and leptin, we then selected the best model based on the DOPE and molpdf scores. The structure of Lep_116-130_ was extracted from the best leptin model (residues 116-130). After the structures were selected and refined, the docking studies were performed using the web server HDock (Yan et al., 2020). The complexes were selected based on whether the interaction between the peptides and LepRb included one of the two potential binding sites for leptin (primary: AA S505, L504, L503; secondary: AA L370, H417, H418, Y420) (Saxton et al., 2023). From HDock, we obtained docking and confidence scores. A more negative docking score indicates a higher binding probability between the proteins in the model. More positive confidence scores indicate a higher likeliness that the two proteins bind. Confidence scores above 0.7 indicate that the two molecules are very likely to bind, whereas scores below 0.5 indicate an unlikely binding (Li et al., 2022).

### 2.4 Cell culture and intracellular calcium imaging

mHypoE-N43/5cells (#CLU127, Cellutions Biosystems) were cultured in Dulbecco’s modified Eagle medium (DMEM) high glucose (#11995-040, Gibco) supplemented with 10% fetal bovine serum (#10437028, Gibco), 100 U/mL penicillin streptomycin (#15140122, Gibco), and maintained at 37 °C with 5% CO_2_ (Hernandez-Caceres et al., 2019). mHypoE-N43/5 cells (100,000 cells) were seeded at ∼50% confluence on 22 mm glass coverslips (#0111620 Marienfield). Once cells reached a confluence of 60%–70%, they were washed with live cell imaging medium (Ozcan et al., 2015). After that, cells were incubated with Fluo4-AM (5 μ M, #F14201 Invitrogen) for 15 min at 37 °C and 5% CO_2_. Covers were gently washed 3 times and transferred into a cell chamber (Attofluor, #A7816, ThermoFisher) to acquire images (1Hz for 600 s) on an epifluorescence microscope (Cell observer Z1, Carl Zeiss AG, Jena, Germany). The first 60 s of basal fluorescence (F0) were used to adjust the experimental fluorescence (F) for the analysis. At 60 s, vehicle, leptin (10 µM), or Lep_116-130_ (10 µM or 100 µM) were added (Ozcan et al., 2015). Carbachol (50 μM, #C4382, Sigma Aldrich) was added between 500 and 550 s as a positive control. For each experiment, cells were identified using the Cellpose algorithm with standard configurations (Stringer et al., 2021), and the algorithm’s cell selection was manually curated in FIJI (v1.54p) to discard false positives. For analysis, the fluorescence was measured in each frame for the whole identified cell.

### 2.5 Analysis of LepRb expression in hypothalamic cell cultures

mHypoE-N43/5 cells (#CLU127, Cellutions Biosystems) were cultured as described in Section 2.4. Total RNA was extracted from cultured cells and total hypothalamus from wild-type mice (RNAEasy Kit, Qiagen) and cDNA was prepared (iScript RT SuperMix, Bio-Rad). Expression of LepRb was assessed by RT-PCR (Fw: 5′-TGG​TCC​CAG​CAG​CTA​TGG​T-3′; Rv: 5′-ACC​CAG​AGA​AGT​TAG​CAC​TGT-3′; Activation: 95 °C × 1 min, Denaturation: 95 °C for 30 s, Annealing: 57 °C for 45 s, Extension: 72 °C for 5 min, 35 cycles; Fast SYBR green Master Mix, Applied Biosystems) and its expression resolved by electrophoresis in agarose gel.

### 2.6 Operant lickometer

The lickometer is a two-pump syringe system installed in an operant behavior cage (MedAssociates ENV-307 A) and has been validated for measuring operant behavior for feeding in mice (Sandoval-Caballero et al., 2024). In this device, mice had access to two equidistant ports located on the same side of the cage, each containing an individual spout connected to a syringe pump. A non-aversive blue light cue located in each port above the spout acts as a cue to indicate whether the spout is active. A light on indicates an active spout (licking can result in the delivery of either ∼5 μL of water or 5% w/v sucrose), and a light off indicates an inactive spout (licking does not result in reinforcer delivery). A capacitive touch sensor (ADA, Adafruit! MPR121) sends a digitized signal that is collected by custom software (implemented on an Arduino Uno) that continuously tracks the number of licks per spout and whether each spout is active or inactive. If the spout is active, a reward is delivered immediately after a mouse performs the required work (i.e., licks the spout a given number of times). After each reward delivery, there is a 20 s period in which the spout is inactive (Sandoval-Caballero et al., 2024).

### 2.7 Sucrose demand test

Mice were trained to consume sucrose (5% w/v) using a 2-port operant lickometer under a fixed-ratio 5 (FR5) schedule for a 5 µL drop of sucrose and water (1 h daily sessions within 2 h from the start of the lights-off period) for 1 week (Sandoval-Caballero et al., 2024). Then, mice performed a demand curve (DC) task (Sandoval-Caballero et al., 2024). In this task, access to water was maintained on an FR5 schedule, while the price of a sucrose reward increased progressively in 10 min intervals (5, 10, 20, 40, 80, and 120 licks) (Sandoval-Caballero et al., 2024). In all tasks, the positions of sucrose and water ports were alternated daily to control for spatial preference.

### 2.8 Subcutaneous Lep_116-130_ infusion

Alzet 1002 mini-osmotic pumps were implanted in *ob*/*ob* mice to deliver either vehicle (sterile PBS) or Lep_116-130_ at a rate of 0.5 mL/h. Sham surgeries were performed in wild-type mice. Surgical procedures for mini-osmotic pump implantation were performed as described (Domingos et al., 2014).

### 2.9 Behavioral measurements

#### 2.9.1 Effect of Lep_116-130_ on intake of standard and palatable food

Wild-type (two males, five females) and *ob*/*ob* (six males, two females) mice were switched from feeding an *ad libitum* standard rodent food to an automated dispenser (FED2) (Koch et al., 2010) that delivers 45 mg pellets (#F0021, BIO-SERV, 3.6 kcal/gr, 20.77% kcal from protein, 13.85% from fat, and 65.37% from carbohydrate). After 2 weeks of acclimation to the FED2, all mice were acclimated to IP injections with daily sham (saline) injections for three consecutive days. Starting on the day after the last sham injection, all mice received IP injections of Lep_116-130_ (1 mg/kg), E4 (10 mg/kg, as a positive control for anorexigenic effects), or vehicle (saline) with at least 48 h between injections and drugs randomized over injection days. All mice received all treatments in a randomized, within-subjects, repeated-measure design. Pellet intake was measured continuously for 24 h after each injection. All the IP injections occurred within 30 min before the lights were off.

Twenty-four hours after the last injection, mice were switched to *ad libitum* standard rodent food for 1 week without any interventions and then trained to consume sucrose-enriched, sweet chocolate-flavored pellets (#F0025, BIO-SERV, 3.83 kcal/gr, 0.26% kcal from protein, 1.3% from fat, 98.44% kcal from carbohydrates) as a model for palatable food from the FED2 for 3 h per day, starting at lights off while maintaining *ad libitum* access to standard rodent food. Then, mice were re-acclimated to IP injections as indicated above. Starting on the day after the third sham injection, all mice received IP injections of Lep_116-130_ (1 mg/kg), E4 (10 mg/kg), or vehicle (saline) with at least 48 h between injections and drug randomized over injection days. Chocolate pellet intake was measured continuously for 3 hours after each injection. All the IP injections occurred within 30 min before the lights were off.

#### 2.9.2 Effect of Lep_116-130_ on glucose tolerance

Wild-type (four males, four females) and *ob/ob* (two males, three females) mice were subjected to a glucose tolerance test (GTT) (Moro and Magnan, 2025). After 6 h of fasting, basal blood glucose was measured with a glucometer (Accu-Check instant #07819374023). Then, mice received an IP injection of vehicle or Lep_116-130_ (1 mg/kg) and 30 min later received a single IP injection of glucose (2 mg/kg). To reduce the number of animals used, we used a cross-over repeated-measures design with mice receiving either saline or Lep_116-130_ with at least 1 week between treatments. For GTT, blood glucose was measured at 15, 30, 45, 60, 90, and 120 min after glucose injection in blood samples from the lateral tail vein (Benede-Ubieto et al., 2020).

#### 2.9.3 Effect of Lep_116-130_ on sucrose demand

Wild-type (three males, two females) and *ob/ob* (six males, two females) mice were trained to conduct daily demand sessions for sucrose for three consecutive days (Sandoval-Caballero et al., 2024). One wild-type mouse was removed because it failed to learn the FR5 task. All remaining mice were included in the experiment. Mini pump implantation surgery was performed in *ob*/*ob* mice to deliver vehicle (PBS, three males and one female) or Lep_116-130_ (three males and one female) (Domingos et al., 2014). Wild-type mice received sham surgery without mini-pump implantation. This design resulted in three experimental groups: wt-sham, *ob/ob*-veh, and *ob/ob*-Lep_116-130_. Between 11 and 13 days after surgery, all mice underwent daily sucrose demand sessions for three consecutive days. Starting on the day of surgery, body weight and food intake were measured daily.

### 2.10 Data and statistical analysis

All data were analyzed using Rv4.4.1. The type-I error rate was set to 0.05 for all statistical tests. For analysis of the effect of Lep_116-130_ on intracellular Ca^+2^, the change in mean intensity of the fluorescence adjusted by the basal fluorescence of each cell was analyzed with a mixed linear regression model, using treatment with experiment (one experiment was equal to one cover, each treated and observed on different days) as a random intercept. Pairwise comparisons were corrected using the Tukey HSD test.

The effects of acute Lep_116-130_ and E4 administration on homeostatic and hedonic intake were analyzed separately for each outcome using a mixed linear regression model for intake, with time (in hours) and genotype (wild-type and *ob/ob*) as fixed effects and individual mice as random intercepts. For homeostatic intake, hour was nested within light periods. The effects of Lep_116-130_ and E4 on the area under the curve (AUC) of the glucose tolerance test were analyzed using a mixed linear regression model for AUC, with treatment and genotype as fixed effects and animal as a random intercept. The effects of chronic delivery of Lep_116-130_ on weight and food intake were analyzed separately using a mixed linear regression model with treatment as a fixed effect and individual mice as a random intercept. For all analyses, pairwise comparisons were corrected with Tukey HSD.

The individual demand data (number of obtained sucrose rewards at each price) for each session was fitted to the Koff equation: log10Q = logQ_0_ + 10^K(e −αQ_0_^ ^C – 1)^ (Koffarnus et al., 2015). From this, we obtained three parameters: Q_0_, α, and Pmax (Koffarnus et al., 2015). Briefly, Q_0_ is the extrapolated consumption when the price required to obtain the reinforcer (i.e., sucrose) is null and has been described as a measure of the hedonic value for the reinforcer used (Bentzley et al., 2013). α is the constant that describes the speed of the decrease in the consumption of sucrose as its cost increases, and is interpreted as the flexibility of the demand for sucrose, such that larger values of α indicate higher flexibility (i.e., consumption decreases faster as the price of the reinforcer increases) (Bentzley et al., 2013). Finally, Pmax is the effort at the maximum events before the demand decays (Koffarnus et al., 2022). We also calculated total sucrose intake during each demand test (Q_T_). The effect of Lep_116-130_ administration on each demand curve parameter, averaged over the second and third demand test sessions, was analyzed with a mixed linear model with the experimental group (wt-sham, *ob/ob*-veh, *ob/ob*-Lep_116-130_) and experimental stage (before or after Lep_116-130_ administration) and their interaction as fixed effects and mice as a random intercept. The effect of genotype (wild-type vs. *ob/ob*) on each demand parameter was analyzed with unpaired t-tests.

## 3 Results

### 3.1 Lep_116-130_ is less likely to bind to the leptin-binding site of the LepRb

**FIGURE 1:**
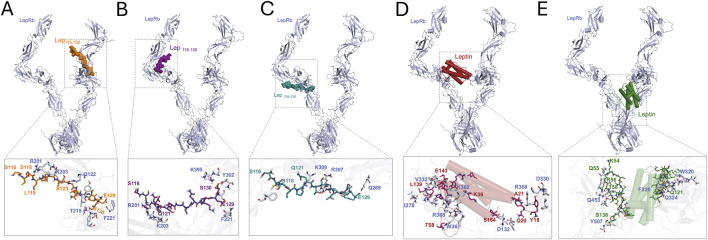
Lep_116-130_ and leptin interact with different sites of the LepRb **(A–C)** 3-D structure of the best three docking complexes between Lep_116-130_ and LepRb, with a close-up to the binding site and interactions. **(D,E)** 3-D structure of the best two docking complexes between leptin and LepRb, with a close-up to the binding site and interactions.

There is conflicting evidence from *in vitro* and *in vivo* studies on whether Lep_116-130_ can bind and activate LepRb. To address this question, we performed *in silico* docking experiments to determine the binding likelihood of Lep_116-130_ to LepRb, compared with that of leptin. After docking, we selected the three best protein complexes between Lep_116-130_ and LepRb and the two best complexes between Leptin and LepRb. The complexes between LepRb and Lep_116-130_ had docking scores of −180.36, −178.82, and −175.32, with corresponding confidence scores of 0.6473, 0.6402, and 0.6240, respectively (Figures 1A–C). On the contrary, the interaction of Leptin and LepRb had lower docking scores of −301.33 and −294.68 and confidence scores of 0.9538 and 0.9475, respectively (Figures 1D,E), indicating that Lep_116-130_ is less likely to bind LepRb than leptin.

The binding of Lep_116-130_ to LepRb in the best complex (docking score = −180.36 and confidence score = 0.6473) was stabilized by interactions with LepRb charged residues K127, E129, and polar residues such as S116, Q126, and S130; all interactions that are not present in the other complexes (Figure 1A). However, the binding of leptin to LepRb in the best complex (docking score = −301.33 and confidence score = 0.9538) was stabilized by different interactions with polar, non-polar, and charged residues (p.e., D132, D330, K362, and R368) from LepRb (Figures 1D,E). Further, compared to Lep_116-130_, leptin interacts with both chains of the receptor, having a larger interaction interface and a greater number of interactions with the LepRb. Altogether, these data suggest that due to fewer interactions and a smaller contact interface, Lep_116-130_ is less likely to bind to LepRb compared to leptin, and that the interaction site between Lep_116-130_ and LepRb differs from that between leptin and LepRb.

### 3.2 Lep_116-130_ does not increase intracellular calcium in a hypothalamic cell line

**FIGURE 2:**
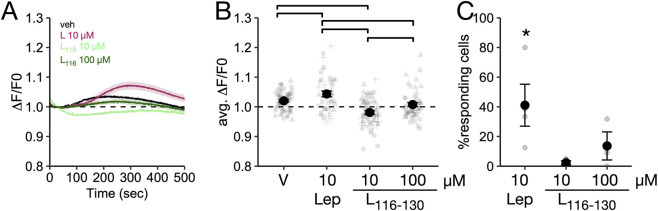
Lep_116-130_ does not increase intracellular calcium in the mHypoN43/5 cell line. **(A)** Mean change in calcium (F/F0) per cell after treatment with vehicle (V), leptin (L) 10µM, or Lep_116-130_ (L_116-130_) at 10 or 100 µM. **(B)** Mean intensity of fluorescence (ΔF/F0) for each cell (points) and per experiment for each treatment. Brackets indicate P < 0.05 for difference between groups. **(C)** Percentage of responsive cells for each treatment. Asterisks indicate P < 0.05 for difference from null. Sample size: V = 3, L = 4, L_116-130_ 10 µM = 4, L_116-130_ 100 µM = 3. Data is presented as mean ± SEM.

Repeated IP Lep_116-130_ administration can reduce food intake and body weight in *ob/ob* and *db/db* female mice (Grasso et al., 1997; Grasso et al., 1999a; Grasso et al., 1999b) and it has been reported that Lep_116-130_ can activate intracellular signaling pathways in the human neuroblastoma cell line SH-SY5Y (Malekizadeh et al., 2017). As the hypothalamus is a key brain region for the control of body weight and food intake (Stuber and Wise, 2016; Rossi and Stuber, 2018; Morton et al., 2014), these data suggest that Lep_116-130_ can alter the activity of hypothalamic neurons, but this has not been directly tested. To evaluate this hypothesis, we examined whether Lep_116-130_ can increase intracellular calcium levels in mHypoN43/5 cells, a hypothalamic non-tumoral-derived cell line that expresses LepRb (Supplementary Figure S1). As shown in Figure 2A, incubation with 10 µM leptin robustly increased average intracellular calcium fluorescence, indicating activation of intracellular signaling (P = 0.017). By contrast, Lep_116-130_ at 10 µM reduced (P = 0.01) or 100 µM failed (P = 0.15) to increase average intracellular calcium fluorescence across all cells compared to vehicle (Figure 2B) and the effect of Lep_116-130_ at 10 and 100 µM was significantly different from that of Leptin (P < 0.001 for both comparisons). Because individual cells showed significant variability in calcium response after incubation with leptin, we quantified the number of cells with intracellular calcium elevations after Lep_116-130_ incubation. Leptin increased intracellular calcium levels in 41.1% ± 14.1% of treated cells (P < 0.001; Figure 2C). By contrast, Lep_116-130_ at 10 and 100 µM increased intracellular calcium signal only in 2.3% ± 1.3% and 13.6% ± 9.5% of the cells, respectively (P = 0.79 and P = 0.2 respectively, Figure 2C). Overall, these data shows that Lep_116-130_ does not increase in intracellular calcium signal in mHypoN43/5 cells.

### 3.3 Acute administration of Lep_116-130_ reduces intake of palatable food, but not intake of standard food, exclusively in *ob*/*ob* mice

**FIGURE 3:**
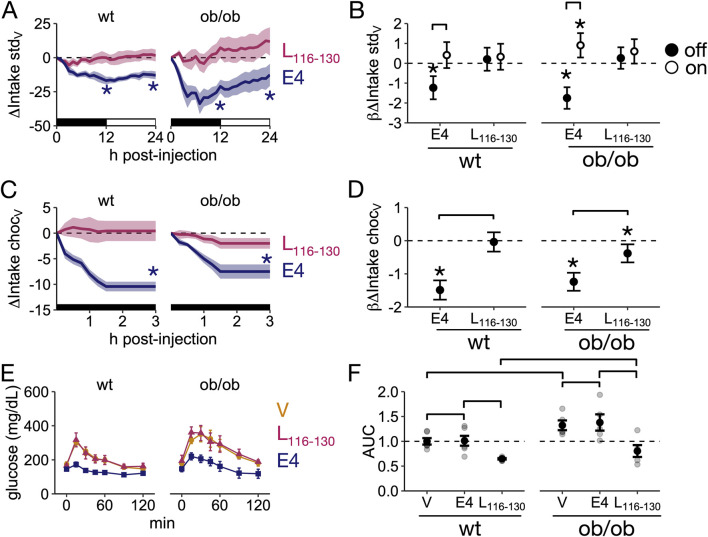
Lep_116-130_ reduces hedonic intake without altering homeostatic intake or glucose tolerance **(A)** Difference in intake of regular chow food for 12 h after a single IP injection of Lep_116-130_ (L_116-130_) or exendin-4 (E4) in wild-type (wt) and *ob/ob* mice compared to a single vehicle IP injection (difference in intake calculated within animals). Black bars: lights off, white bars: lights on. **(B)** Slopes of change in food intake over hours relative to vehicle after E4 and Lep_116-130_ IP administration during lights-off and lights-on. **(C)** Difference in intake of sweet chocolate pellet intake for 3 h after a single IP injection of Lep_116-130_ or E4 in wild-type and *ob/ob* mice compared to vehicle injection (difference in intake calculated within animals). **(D)** Slopes of change in sweet palatable intake over hours relative to vehicle after E4 and Lep_116-130_ IP administration. **(E)** Glucose tolerance test after a single IP injection of vehicle, Lep_116-130,_ or E4 in wild-type and *ob/ob* mice. **(F)** Area under the curve (AUC) of glycemia for each treatment. **(A–D)**: wild-type, n = 7; *ob/ob,* n = 8. **(E,F)**: wild-type, n = 8; *ob/ob, n* = 5. **(B,D)** Data is expressed as estimated marginal slope ±95%CI. **(E,F)** Data is expressed as mean ± SEM. Panels **(B,D,F)**: Brackets indicate P < 0.05 for pairwise comparisons. Panels **(B,D)**: Asterisks indicate P < 0.05 for difference from null.

It has been reported that repeated daily Lep_116-130_ administration reduces food intake and body weight in *ob*/*ob* and *db*/*db* female mice (Grasso et al., 1997; Grasso et al., 1999a; Grasso et al., 1999b); however, whether acute administration of Lep_116-130_ can reduce homeostatic and hedonic intake is unclear. Further, quantifying only total daily food intake does not capture the full effects of Lep_116-130_ on food intake. Thus, we characterized the time course of homeostatic and hedonic food intake after Lep_116-130_ treatment by measuring the change, compared to vehicle, in the intake of standard and sweet chocolate-flavored food pellets in wild-type and *ob*/*ob* mice. As a positive control for acutely decreased food intake, we used Exendin-4 (E4), a GLP1R agonist that crosses the blood-brain barrier with potent anorexigenic actions (Baraboi et al., 2011). We first tested whether Lep_116-130_ can reduce homeostatic food intake by measuring intake of standard rodent pellets 24 h after a single IP Lep_116-130_ administration. First, as expected, the overall 24-h intake after vehicle injection was greater in *ob*/*ob* mice compared to wild-type mice (Supplementary Figure S2). While E4 reduced intake of standard rodent pellets across at 12 h and 24 h post injection in wild-type (12 h, P = 0.0013; 24 h, P = 0.023) and *ob/ob* mice (12 h, P < 0.0001; 24 h, P = 0.0008), Lep_116-130_ did not modify the intake of standard rodent pellets either in wild-type or *ob/ob* either 12 h or 24 h after injection (P > 0.05 for all endpoints, Figure 3A). The slope for food intake over time after E4 administration in the lights-off period was negative and significantly different from zero for both wild-type and *ob/ob* mice (P < 0.0001 for both genotypes), and consistent with its lack of effects on overall intake, the slope after Lep_116-130_ administration was not different from zero for wild-type (P = 0.4) and *ob/ob* mice (P = 0.34; Figure 3B). We next tested whether Lep_116-130_ can reduce hedonic intake by measuring the intake of palatable, sweet-chocolate pellets for 3 h after a single IP Lep_116-130_ administration. As a positive control, we found that a single IP administration of E4 reduced chocolate pellet intake 3 h after injection in wild-type and *ob/ob* mice (P < 0.001 for both genotypes, Figure 3C) resulting in negative slopes of change in the intake curves of chocolate pellets in wild-type and *ob/ob* mice (P < 0.001 for both genotypes, Figure 3D). Interestingly, whereas the administration of Lep_116-130_ had no effects in wild-type mice 3 h after injection (P = 0.56), it led to a reduction (Figure 3C) and a significantly negative slope for intake of sweet-chocolate pellets in *ob*/*ob* mice (P = 0.006; Figure 3D). Together, these data indicate that a single IP administration of Lep_116-130_ does not reduce homeostatic food intake in wild-type or *ob*/*ob* mice, but it can selectively reduce intake of sweet, chocolate-flavored pellets in *ob*/*ob* mice.

Because leptin reverses insulin resistance and glucose intolerance in *ob*/*ob* mice independently of their effects on food intake (Koch et al., 2010), we also assessed the effects of Lep_116-130_ on glucose tolerance in wild-type and *ob*/*ob* mice. As expected, *ob*/*ob* mice had a lower glucose tolerance compared to wild-type mice, as indicated by the larger area under the curve during the GTT (P = 0.03; Figures 3E,F). While the administration of a single IP injection of E4 significantly improved glucose tolerance in wild-type (P = 0.02) and *ob/ob* mice (P = 0.002, Figures 3F), a single IP injection of Lep_116-130_ failed to modify glucose tolerance in wild-type and *ob/ob* mice (P > 0.05 for both genotypes, Figures 3E,F).

### 3.4 Chronic administration of Lep_116-130_ reduces demand for sucrose in *ob*/*ob* mice

**FIGURE 4:**
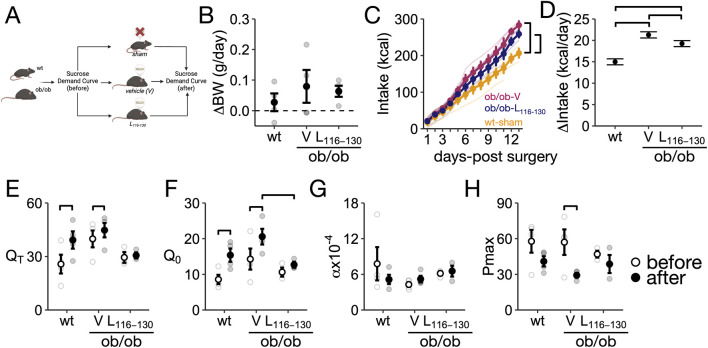
Chronic administration of Lep_116-130_ to *ob/ob* mice decreases motivation for sucrose intake. **(A)** Experimental design: demand for sucrose was measured before and after chronic treatment (14 days) with Lep_116-130_ or vehicle in *ob/ob* mice. A group of wild-type (wt) mice subjected to sham surgery was included as a control. **(B)** Daily change in body weight from surgery to the end of the experiment. **(C)** Cumulative food intake after surgery. **(D)** Rate of cumulative food intake after surgery. **(E)** Total intake of sucrose during the demand test (Q_T_). **(F)** Demand intensity (Q_0_). **(G)** Demand flexibility ( α ) **(H)** Pmax. Group size: wild-type-sham = 4, *ob/ob* V = 4, *ob/ob* Lep_116-130_ = 4. Data is expressed as mean ± SEM. Panels **(C,D)**: Brackets indicate P < 0.05 for pairwise comparisons between groups. Panels **(E,H)**: Brackets indicate P < 0.05 between groups and time periods.

The selective effects of Lep_116-130_ on the intake of sweet chocolate-flavored pellets in *ob*/*ob* mice led us to investigate whether Lep_116-130_ can modulate motivated behaviors underlying hedonic intake in *ob/ob* mice. Thus, we examined changes in sucrose demand before and after long-term delivery of Lep_116-130_ (0.25 μ g/h, 1 mg/day subcutaneously for 2 weeks) or vehicle in *ob*/*ob* mice by using subcutaneous osmotic mini-pumps. As control, we included wild-type mice subjected to sham surgeries (wt-sham; Figure 4A).

Mice from all groups gained weight during the experiment, but there were no differences in body weight change between *ob*/*ob* mice from both groups after completing the experiment (P > 0.05 between all groups; Figure 4B). Expectedly, *ob*/*ob* mice from both groups had a larger body weight compared to wild-type mice (*ob/ob*-Lep_116-130_: 53.4 ± 0.64 g; *ob/ob*-V: 53.9 ± 2.26 g; wt-sham: 25.9 ± 0.41 g; P < 0.05 for comparisons between *ob/ob*-Lep_116-130_ and *ob/ob*-V to wt-sham mice). Cumulative food intake was not different between *ob*/*ob* mice from either group (P = 0.19), but *ob*/*ob* mice from both groups had a larger total cumulative intake compared to wt-sham mice (P < 0.05 for each comparison). To further examine the effect of Lep_116-130_ on intake, we examined the rate of food intake over the duration of the experiment (Figure 4C). Lep_116-130_ administration reduced the rate of food intake in *ob*/*ob* compared to vehicle administration (P = 0.003), and as expected, *ob*/*ob* mice administered vehicle or Lep_116-130_ had a larger food intake rate compared to wt-sham mice (P < 0.001 for both comparisons, Figure 4D).

To determine whether long-term administration of Lep_116-130_ modified motivated behaviors for palatable food, mice from the three groups (wt-sham, *ob/ob*-Lep_116-130_, *ob/ob*-V) were tested on a sucrose demand task before surgery and after 2 weeks of treatment. Compared to the pre-surgery status, wt-sham showed increased total sucrose intake (Q_T_, P < 0.001) and sucrose demand intensity (Q_0_, P < 0.001) with no significant changes in sucrose demand flexibility ( α , P = 0.08) or Pmax (P = 0.11, Figures 4E–H). However, compared to the before surgery, *ob/ob*-V showed a significant increase in total sucrose intake (Q_T_, P = 0.007) and demand intensity (Q_0_, P = 0.001) and a significant reduction in Pmax (P = 0.01) without changes in demand flexibility (P = 0.51, Figures 4E–H), whereas *ob/ob*-Lep_116-130_ did not show significant changes in any of the sucrose demand parameters (P > 0.05 for all parameters, Figures 4E–H). Further, the sucrose demand intensity (Q_0_) after 2 weeks of treatment was significantly lower in *ob/ob*-Lep_116-130_ compared to *ob/ob*-V (P = 0.05, Figure 4F). Together, these data suggest that chronic Lep_116-130_ administration reduces the rate of food intake, prevents increases in sucrose intake (Q_T_) and demand intensity (Q_0_), and reduced flexibility (lower Pmax) in *ob*/*ob* mice.

## 4 Discussion

In this study, we aimed to gain insights into the biological effects of Lep_116-130_ and determine whether this peptide can reduce hedonic intake. We present three key findings. First, Lep_116-130_ does not likely bind to LepR and does not activate signaling in hypothalamic neurons. Second, in *ob*/*ob* mice but not in wild-type mice, acute administration of Lep_116-130_ (1 mg/kg IP) reduced the rate of food intake and the overall amount of sweet-chocolate pellets consumed, without reducing the rate of intake or the overall amount of standard rodent pellets consumed. Third, compared to vehicle administration, chronic administration of Lep_116-130_ to *ob*/*ob* mice prevented behavioral adaptations to the repeated availability of sucrose in a demand task, while reducing the rate of standard pellet intake, without inducing significant differences in body weight after 2 weeks of treatment. Together, these data indicate that Lep_116-130_ can reduce hedonic intake and motivational aspects of palatable food intake, suggesting the potential of this synthetic peptide as a tool to manipulate hedonic intake.

Our data showing that Lep_116-130_ can reduce intake of palatable food is consistent with data showing that repeated IP administration of Lep_116-130_ (28 days at 1 mg/day) reduced body weight by 3.43% and food intake by ∼14% in female *ob/ob* mice (Grasso et al., 1997) and blocked the increase in body weight in female *ob/ob* mice starting 24 h after the treatment (Grasso et al., 1999b). Methodological differences can explain the discrepancy between our results and those of previous studies, including dose, timing of injection relative to the circadian cycle, continuous vs. intermittent food access, and sex (in our studies, we combined males and females, while previous feeding studies used only females (Grasso et al., 1997; Grasso et al., 1999b)). Herein, we measured intake continuously during the 24 h after a single IP injection of Lep_116-130_, without observing any circadian-dependent effect. In our chronic studies, we employed a duration of administration similar to that used in studies reporting the effects of leptin on body weight and food intake in mice (Halaas et al., 1995; Wong et al., 2016). Not observing an effect of Lep_116-130_ on wild-type mice is suggestive, as one of the key characteristics of *ob*/*ob* mice is their increased sensitivity to exogenous leptin (Harris et al., 1998), and connectome brain and neuroendocrine remodeling that alters their food intake control and reward responses (Perez-Leighton et al., 2024). While testing whether Lep_116-130_ can indeed reduce food intake and reward in wild-type mice requires additional studies, our results suggest that Lep_116-130_ preferentially reduces hedonic intake in a background of leptin deficiency, suggesting a specific effect of Lep_116-130_ on reward behavior.

We observed that compared to their basal condition (before surgery), both wt-sham and *ob/ob* mice treated with vehicle showed increased total intake of sucrose (Q_T_) and the intensity of sucrose demand (Q_0_) during sucrose demand sessions. These behavioral changes are consistent with the increase in intake reported in different tests of palatable food intake under intermittent access in mice and rats (Awad et al., 2024; Yasoshima and Shimura, 2015; Wojnicki et al., 2007). The reduction in Pmax observed in *ob/ob* mice is more puzzling, as a lower Pmax indicates a reduction in the maximum price before initial sucrose intake is reduced (i.e., the animal becomes more sensitive to increased sucrose prices). The *ob*/*ob* mice have a higher reward value for sucrose, yet they do not show increased sucrose preference under free-access conditions (Domingos et al., 2014; Muelbl et al., 2016), which is consistent with increased demand intensity (Q_0_, intake at low sucrose price) but reduced Pmax (less willingness to maintain their intake as price increases). Our finding that Lep_116-130_ blocked all these behavioral adaptations, without significant changes in body weight, warrants further exploration of the peptide’s capabilities to influence food reward behaviors through its action in the brain.

Our molecular docking and *in vitro* studies suggest that LepRb might not fully mediate the actions of Lep_116-130_ on food intake. While we lack the definite experiment testing Lep_116-130_ in the absence of LepRb, we present three lines of evidence suggesting that LepRb is not the major receptor for Lep_116-130_. First, our molecular docking analyses show that Lep_116-130_ interacts with LepRb in different sites than full-length leptin, and it has a very low probability of binding to one of the three canonical sites required for LepRb activation (Fan et al., 2023). Second, our *in vitro* experiments showed that Lep_116-130_ did not increase in intracellular calcium concentration. These data are consistent with evidence showing that Lep_116-130_ does not bind to the LepRb expressed in COS-7 and GT-1 cell lines (Grasso et al., 1999b). Third, the notion that Lep_116-130_ does not act through LepRb is consistent with our data showing that injection of Lep_116-130_ did not increase glucose tolerance in *ob/ob* or wild-type mice. Because leptin potently reduces homeostatic intake and glucose intolerance in *ob/ob* mice (Halaas et al., 1995; Wong et al., 2016), these findings suggest that the activation of LepRb is necessary for regulating hunger and satiety (processes largely regulated by the hypothalamus) (Perez-Leighton et al., 2024) and glucose tolerance in both wild-type and *ob*/*ob* mice. Thus, while we cannot exclude that LepRb contributes to the biological effects of Lep_116-130_, our data are consistent with the interpretation that LepRb is not the primary molecular target for Lep_116-130_.

A limitation of our study is that we did not conduct an extensive dose-response curve for the effects of Lep_116-130_ on our behavioral tests. This study remains necessary because translational relevance of the use of Lep_116-130_ remains understanding the effective minimal dose, ceiling and potential side effects of chronic and acute Lep_116-130_ administration. While in our molecular docking and cellular studies we included leptin as a control, we decided not to include leptin and instead included EX4, a GLP1R agonist, as a control in our acute administration studies. Our rationale was not to compare Lep_116-130_ against leptin, because the goal of this paper was not to determine whether Lep_116-130_ can replicate the anorexigenic effects of full leptin, but rather to have a potent and rapid positive control for an anorexigenic effect in wild-type and *ob*/*ob* mice. For our chronic administration studies, the study was designed to test the effects of Lep_116-130_, regardless of whether the effects are similar to those of leptin. Thus, we decided not to include leptin as a control in the study. Finally, we included male and female mice in our study because our goal was to establish an effect of Lep_116-130_ on eating behavior regardless of sex and hormonal influences, consistent with evidence indicating that variability across different behavioral endpoints is not different between females and males (Beery, 2018; Becker et al., 2016; Prendergast et al., 2014; Kaluve et al., 2022). Relevant to the influence of sex differences on Lep_116-130_ effects, *ob/ob* mice are sterile due to undeveloped gonads, leading to lower levels of sex hormones and sterility in both sexes (Mounzih et al., 1997; Chehab et al., 1996). Thus, our study was not powered to detect sex differences and whether sex impacts the effects of Lep_116-130_ on eating behavior remains to be determined.

The translational potential of our findings lies in the fact that common human obesity can be largely explained by excessive hedonic intake (Krebs, 2009; Drewnowski et al., 2020; Collaborators, 2019); thus, an ideal therapeutic tool should selectively target hedonic intake without affecting homeostatic and nutritional regulation. Also, dysregulated hedonic feeding is central to several eating behavior disorders, such as binge eating disorder and bulimia nervosa, where patients may frequently present with either normal, low, or even increased body weight (Grilo, 2024). In such contexts, agents capable of attenuating reward-driven food consumption without altering homeostatic feeding and without important gastrointestinal side effects, such as GLP1, could represent a novel therapeutic avenue. Thus, the ability of Lep_116-130_ to reduce hedonic but not homeostatic intake positions this peptide as an interesting alternative to treat both obesity and eating behavior disorders.

## 5 Conclusion

Our data indicate that Lep_116-130_ does not affect homeostatic intake in either wild-type or *ob*/*ob* mice; however, it selectively reduces consumption of sweet, palatable foods and prevents behavioral adaptations to repeated operant sucrose exposure in *ob*/*ob* mice. Together with our *in silico* and cellular findings, these results provide a proof-of-concept that, likely without a major contribution of LepRb binding, Lep_116-130_ can regulate hedonic food intake.

## Data Availability

Original data will be made available on request. The names of the repository/repositories and accession number(s) for public data used in this study can be found below http://www.wwpdb.org/, PDB 8DH8, http://www.wwpdb.org/, PDB 1AX8, https://www.ncbi.nlm.nih.gov/genbank/, ADM72802.1.
