# Supplementary material for: The leptin fragment Lep116-130 attenuates hedonic consumption and sucrose-seeking in mice
Source: Front Pharmacol. 2026 Mar 24;17:1748508. doi: 10.3389/fphar.2026.1748508 (PMC13054474; doi:10.3389/fphar.2026.1748508)
Supplement: Supplementary file 1 [file DataSheet1.docx]

**Supplementary Figures**


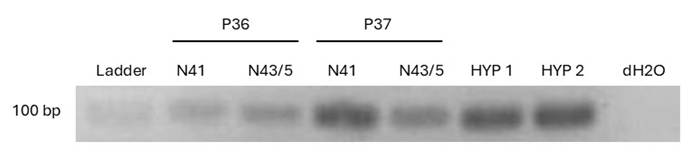


**Supplementary figure 1. mRNA expression of LepRb in hypothalamic cell cultures.** RT- PCR for LepRb of mHypoN41and mHypoN43/5 cell line in passages 36 and 37. Two hypothalamuses of WT mice were used as control. dH2O plus primers was used as a negative control for the technique. mHypoN43/5 (#CLU127, Cellutions Inc) were used a positive control for expression of LepRb and were cultured as described in Methods.


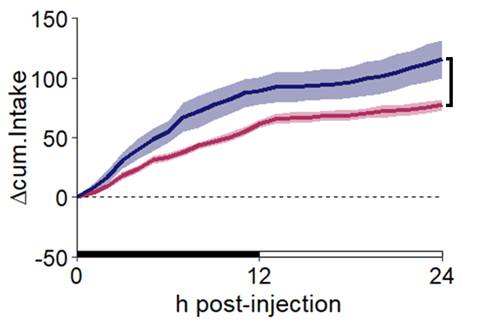


**Supplementary figure 2. Cumulative 24-hour homeostatic intake of WT and *ob/ob* mice.**

Cumulative food intake of wild- and *ob/ob* after the injection with vehicle (saline). WT, n = 7; *ob/ob,* n = 8. Data is shown as mean±SEM. Bracket, P<0.05 for pairwise comparisons.
